# The subconscious impact of line orientations in background images on memory of Chinese written characters

**DOI:** 10.1371/journal.pone.0269255

**Published:** 2022-05-31

**Authors:** Yanqun Huang, Yi Zhang, Xu Li, Jie Zhang, Yuzhen Wang

**Affiliations:** 1 Key Laboratory of Mechanism Theory and Equipment Design of Ministry of Education, Tianjin University, Tianjin, China; 2 Tianjin Ren’ai College, Tianjin, China; 3 Tiangong University, School of Mechanical Engineering, Tianjin, China; 4 Tianjin Sino-German University of Applied Sciences, Tianjin, China; Northeastern University, UNITED STATES

## Abstract

Oriented lines impact human cognition subconsciously. This study aimed to determine whether line orientations in the background of Chinese written characters influenced individual’s memory and emotion. Five pictures with Chinese characters as experimental material, in which four had equidistant parallel lines (0°, -45°, 90°, and +45°) as background and the other one had a blank background, were presented on a personal computer screen, for 15 seconds each, to 94 participants. The participants were then given 45 seconds to write down what they had just memorized. Participants’ emotion was identified by their Heart Rate Variability (HRV) simultaneously during the viewing process. The results showed that vertical (90°) and 45° leftward leaning lines (-45°) did not weaken users’ memory, and no significant difference in memory was found between these two states and the blank background, while horizontal (0°) and 45° rightward leaning lines (+45°) weakened the memory effect significantly. Overall, memory decreased in the condition of horizontally lined background while no influence in vertically lined background condition; and it showed asymmetry under leftward and rightward leaning line conditions: memory decreased in rightward leaning lined background while no influence in leftward lined background. Moreover, the results of emotion and memory showed negative similar trend. These findings provide practical suggestions for visual information design.

## Introduction

In ancient Asian culture, people believe that Fengshui is a decisive factor of residents’ wealth, fortune and flourishing [1], while orientation is one of the key elements of Fengshui. Line orientations in Fengshui are often associated with the directionality of mountains, rivers, roads, buildings and even furniture [2, 3], and are considered to influence the prosperity of the individual. The laws of Fengshui have still been widely used today in architecture in Asian countries [4–6]. However, it remains mysterious why orientation plays a role and how it affects people’s life. People just follow some established rules in the relevant design, such as the road should not go straight toward the house, etc. These cultures provoke thinking that: does directionality affect people’s life with subconsciously influenced people’s psychological status and behavior?

In modern science, researchers found that oriented lines or objects contribute significantly to visual perceptions [7, 8]. Under certain circumstances, the lines and line orientations in an image affect people’s perception, behavior, and even decision-making [9–11]. Studies have shown that different line orientations result in different effectiveness in visual texture segregation [12], and in commercial packaging design, graphical elements such as textures evoke emotional associations [13].

Therefore, it is of interest to know that how orientation affects our emotion and cognition subconsciously in one of today’s dominant activities, the interaction between human and computer interfaces. Many people spend plenty of time on communicating with computer and smartphone displaying interfaces, and deal with a large number of 2D graphics in their daily life. The elemental compositions of 2D graphics consist of points, lines and planes. Therefore, there comes questions as: does the lines expressing directionality affect people’s memory and emotion? How does it affect? Aiming to explore answers to the questions, this study conducted an experiment on individual’s memory performance and emotional valence effect of Chinese written characters, with different line orientations as background image.

## Literature review

Recent theoretical developments have revealed that line orientations are fundamental cues for human emotion [14], perception and cognition. For example, regularly repeated horizontal /vertical lines make positive impressions as favorite, tidy, and bright, while oblique lines make negative impressions as gloomy, cold and complicated [15]. In architecture, the oblique lines provide kinesthetic perception and motion [16]. Meanwhile, concave, convex, or straight lines are often associated with happiness or sadness [17]. In some cases, spirals express negative emotions, and twirls show confusion and dizziness [18]. The visuospatial bias correlates with human emotion [19]. Thus, designers like to clearly express characters’ emotion by placing lines appropriately in the scenes of an animation [20]. Besides, user visual attention and memory are more sensitive to stripes with clear contours than to stripes without obvious boundaries under the same conditions, because human vision can clearly recognize the direction of stripes [21]. People perceive horizontal and vertical orientations more easily than oblique oriented lines [11, 22, 23], and often overestimate the length of a vertical line versus a horizontal line [11]. Some studies have shown that the contour information and surface features of an object can be encoded separately when performing visual memory tasks, and memory capacity is larger when operating on objects with boundary lines than with other surface features [21]. Line orientations also affect human behavior, e.g. the drawing stability for the elderly people is much higher for the top-down direction than for the bottom-up direction [24]. Therefore, users’ emotion, perception and cognition depend on but differ from directions. Additionally, studies showed that the higher level of people’s emotion, the better the memory is [25]. Madan et al. reported that positive emotion enhances memory [26], while in other situations, negative emotion helps mnemonic precision [27, 28].

Meanwhile, from perspectives of culture [29, 30] and neuroscience research progress [31], user image perception and directionality preference are affected by the reading habit [32]. Reading habits also cause asymmetry in nonverbal behaviors [31, 33], for example, the judgement of bisection lines [31, 33, 34], the preference of facing direction of objects [30], and even drawing behavior [35]. Left-to-right readers prefer things with left-to-right directionality [36] and have an aesthetic preference for images that face rightward [29]. Thus, the visual asymmetry also comes from people’s behavioral habits. However, for hand laterality, studies have proved that there is no impact on visual perception [37] or emotion [38].

In visual design, especially information design in user interaction, core information could be remembered and understood differently by using certain background pictures. Background information increasement enriches user’s visual experience; furthermore, it ensures that when the foreground object appears in an appropriate position in a relevant scene, the visual search or recognition process is quicker. This phenomenon is called the “context effect” [39], and is often applied as a design method in real life. For example, using a background image related to a product in an advertisement helps consumers understand the thematic information and thus increases sales [40]. This also confirms that the correlation between two objects is a potential factor for changing visual short-term memory effectiveness [41]. However, to ensure the simplicity of a picture in practical applications, other elements such as lines, textures, and color blocks which are unrelated to the subject are often used as a background. Researchers found that elements of a picture unrelated to the subject affect a viewer’s perception subconsciously. Unconscious and conscious memories combine to play a unique and complementary role in guiding an individual’s visual attention to facilitate the effective processing of information [42, 43].

In summary, researchers have affirmed that the influence of line direction and background image on people’s cognition, emotion and behavior cannot be ignored. Especially, people perceive differently for distinct orientations [11, 22, 23], and reading habit does have influence on people’s image perception [32]. However, limited open literature has reported on how line orientations in a background picture affect emotion and cognition subconsciously. Therefore, this study selected four different line-orientation images as different background with Chinese written characters, as research materials, and explored subconscious impact of line orientations on memory of quadrate pictographic textual information.

## Hypothesis

We hypothesized that different line orientations in the background image have different effects on individuals’ memory and emotion, with full-screen displayed and equidistantly arranged.

The independent variable was the line orientation in the background, while the dependent variables were users’ memory performance and emotion index, where SDNN (Standard deviation of Normal to Normal intervals) of HRV (Heart Rate Variability) was used to identify participants’ emotion according to present researches [44, 45].

## Methodology

This study was approved by the Ethics Committee of Tianjin University and the experiment was performed in accordance with approved guidelines. Every participant signed a written informed consent for inclusion prior to the experiment.

The experiment was performed in a controlled environment with identical equipment settings, by presenting random orders of visual stimuli groups (Chinese characters) to balance the potential effect of displaying sequence. The impact of a certain number of arranged oriented lines in the text’s background on participants’ memory and HRV was tested. The recall method was used according to previous experiment [46].

### Participants

A total of 94 college students participated in this study. They were left-to-right readers and right-handed users. All participants had passed the Chinese test in China’s College Entrance Examination, which means they had the required abilities in reading or writing modern Chinese characters. They were typical users of 2D graphics, such as websites and other electronic interfaces. The ratio of male to female was 1:1, and participants’ ages ranged from 18 to 30 years. They had normal visual, reading, and cognitive abilities. Prior to the experiment, each participant had sufficient rest and self-reported that he/she was at normal emotional state and without taking any medicine or alcohol.

### Stimuli

**Fig 1:**
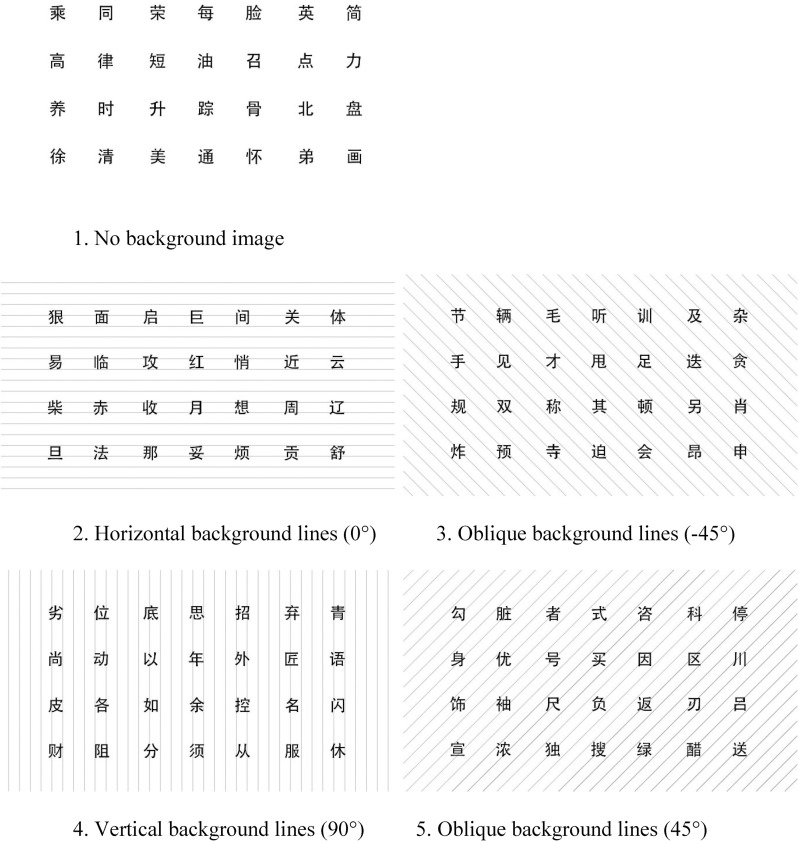
Stimuli.

To measure the impact of distinct background line orientations on memory effectiveness, five pictures of 960 × 720 px were used as stimuli in the experiment (Fig 1). In one picture, the background was a blank white-colored image. In the other four pictures, the background was made up of parallel lines arranged at equal intervals. The content of the foreground was 28 different Chinese characters in each picture, the English translation of which is shown in S1 Table. To ensure that there were no significant cognitive differences between the five groups of Chinese characters stimuli, the characters were balanced by three steps as follows.

First, selection of the stimuli. The Chinese characters were generated on an online Word Generator [47], which picked up words that are commonly used on a daily basis; characters were also taken from a manual of the Basic Vocabulary Table of Modern Chinese Characters [48]. The selected characters are popular for Chinese speakers and can be recognized by those who have finished a basic junior middle school education in China. According to existing research, positive words imply positive emotion [49] and extremely positive characters improves cognition [50]. Especially, positive words can be learned more efficiently, whereas negative and neutral words do not impact on user cognition [51]. Therefore, in this experiment, extremely positive characters were not used, to avoid the influence from the characters. These characters were not easy to confuse, had no textual coherence, and could not be associated with specific contextual meanings.

The pictures’ height in Fig 1 was as same as that of the computer screen when shown to viewers.

Second, interference adjustment. Chinese characters are originated from quadrate pictographic symbols, which are composed of structural strokes. There are several strokes with different orientation in Chinese characters, e.g. horizontal strokes as “-”, vertical as “|”, +45° leaning as “╯” and -45° leaning as “╰”, which could be interfered by the oriented lines in the background. Therefore, the characters in the list were adjusted manually according to the strokes direction included in each character, to avoid the situation of interference and to reach even distribution roughly in the five pictures.

Third, pilot test. The test was performed to ensure that there were no significant differences in the effects on memory among the five groups of characters. In the pilot experiment, we recruited 29 college student volunteers, besides the 94 participants who joined the formal experiment. They were asked to memorize the characters against a blank white background, which was presented for 15 seconds, during which time the participants reported they could read through the stimulant characters; then, they wrote down what they recalled in 45 seconds. Results showed that there were no significant differences between the five verbal stimuli groups (*df* = 4, *F* = 0.037, p = 0.997 (p>0.05)), which indicated that the stimuli used in this study had equal influence on memory.

### Experimental procedure

The formal experiment was conducted on each participant individually, and lasted about 8 minutes each, including preparation and testing (e.g., Electrocardiograph equipping, stimuli presenting, memory, writing, and waiting time). Two assistants collected the raw results after the process.

*Step 1—Preparation*: The experiment was conducted in a controlled environment with identical equipment settings: consistent illumination, sound insulation, and room temperature. There was a laptop and piece of paper on a desk and a chair in front of the desk. Before the experiment, each participant was asked to adjust to a comfortable sitting position at an appropriate viewing distance, and the first 30 of the participants, 15 females and 15 males, were equipped with an Electrocardiograph (provided by KingFar Limited company) for HRV measurement.

First, the participants were informed of the general process by both oral and written notification in Chinese, the English translation of which was as follows:

You will receive five pictures containing some common Chinese characters. You need to memorize as many characters as you can from each picture. Each picture will appear for only 15 seconds and then disappear. There will then be 45 seconds for you to write down the memorized characters. The order is not important in the recall. After that, you will have a five second break before the next round. The process will be repeated five times before coming to an end. Once the process is finished, hand over your writing paper to the assistants.

The notification emphasized that the purpose of the task was to test memory, without mentioning the background information.

**Fig 2:**
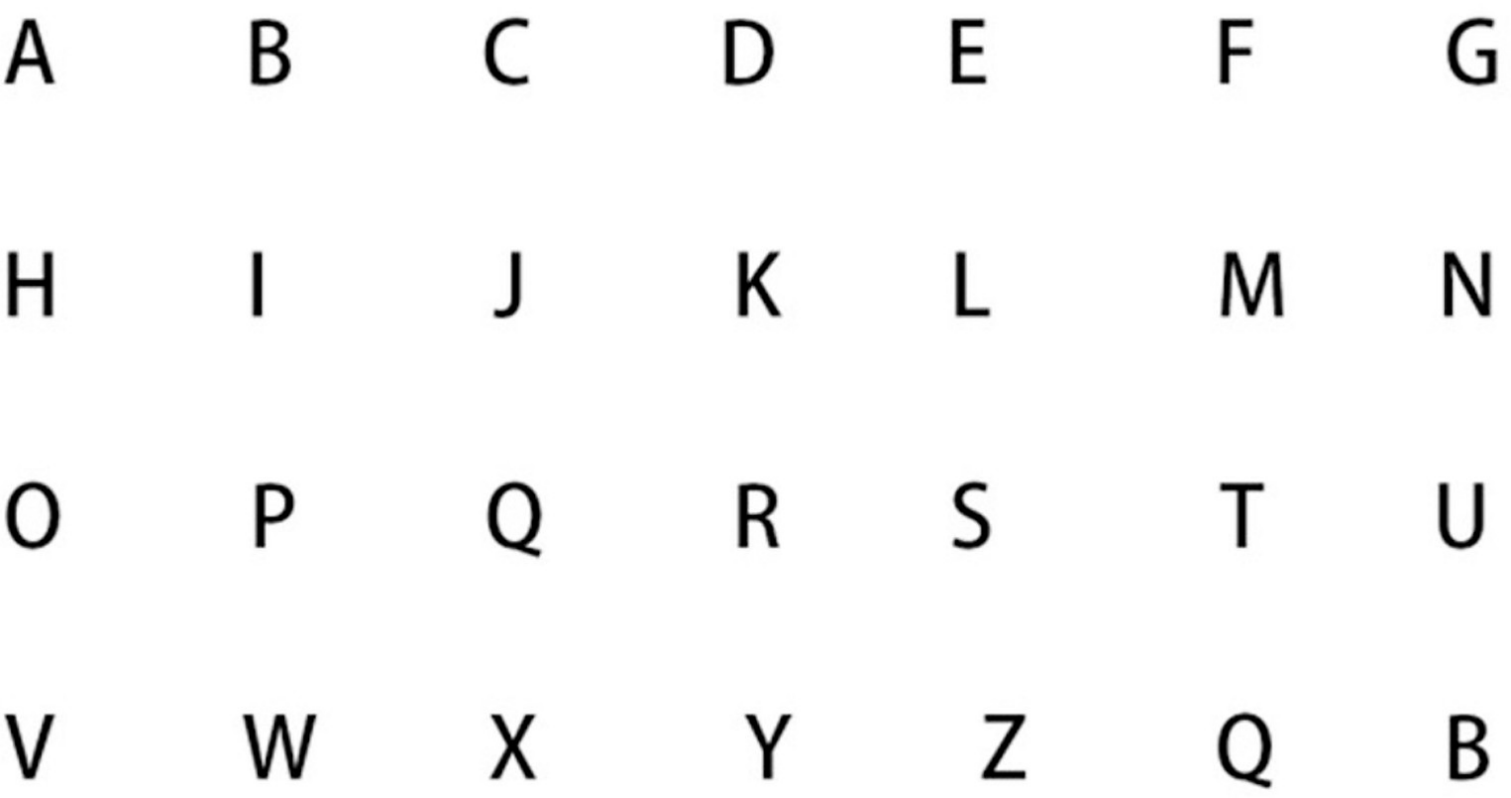
The sample picture used to inform participants of the experiment procedure before memory testing.

Second, a sample picture with some English letters was shown to each participant to inform them of the process and get them used to the duration of each round, to minimize feelings of nervousness and unpreparedness (Fig 2). The English letters were not used to interfere with the Chinese characters in testing, nor to increase the memory burden on participants.

*Step 2 –testing*: When the task began, the fixation signal “+” was shown on the center of the screen to focus the participant’s attention. Following that, five slides with Chinese characters were presented to each participant in a random order for 15 seconds; for example, for one participant, the showing order could be: Picture 1 → Picture 5 → Picture 4 → Picture 2 → Picture 3 (Fig 1), while for another participant: Picture 2 → Picture 3 → Picture 5 → Picture 4 → Picture 1 (Fig 1). For each picture, the participants wrote their answers down within 45 seconds. Meantime, their HRV were recorded.

When completing the memory test, each participant was asked three questions in Chinese; the English translation for these were as follows.

1. Did you notice the background lines in each picture? If yes, please write down a ‘△’ on your answer sheet.

2. Were you aware of the variation in line orientations in the background of each picture? If yes, please write down a ‘√’ on your answer sheet.

3. Do you think line orientations influence memory capacity? If yes, please write down a ‘○’ on your answer sheet.

After the experiment, the participants were informed of its real purpose.

## Results

There were only 92 answer sheets were collected, because two participants’ handwriting were difficult to decipher. After verifying the experimental data based on the PauTa criterion [52], to ensure that the data values of each group were within the range of mean ±3σ and passing the homogeneity variance test, 91 valid sets of data were obtained and analyzed using Analysis of Variance (ANOVA).

### Subjective responses

Participants’ answers showed that about 1/2 of them focused on the foreground Chinese characters as they were asked to memorize, and 36 participants (39.1%) did not notice the background information or the variation in line orientations. The questionnaire results showed that only two out of 92 participants thought line orientations influenced their memory performance, which indicated that the background lines influenced the result subconsciously.

### Memory

**Fig 3:**
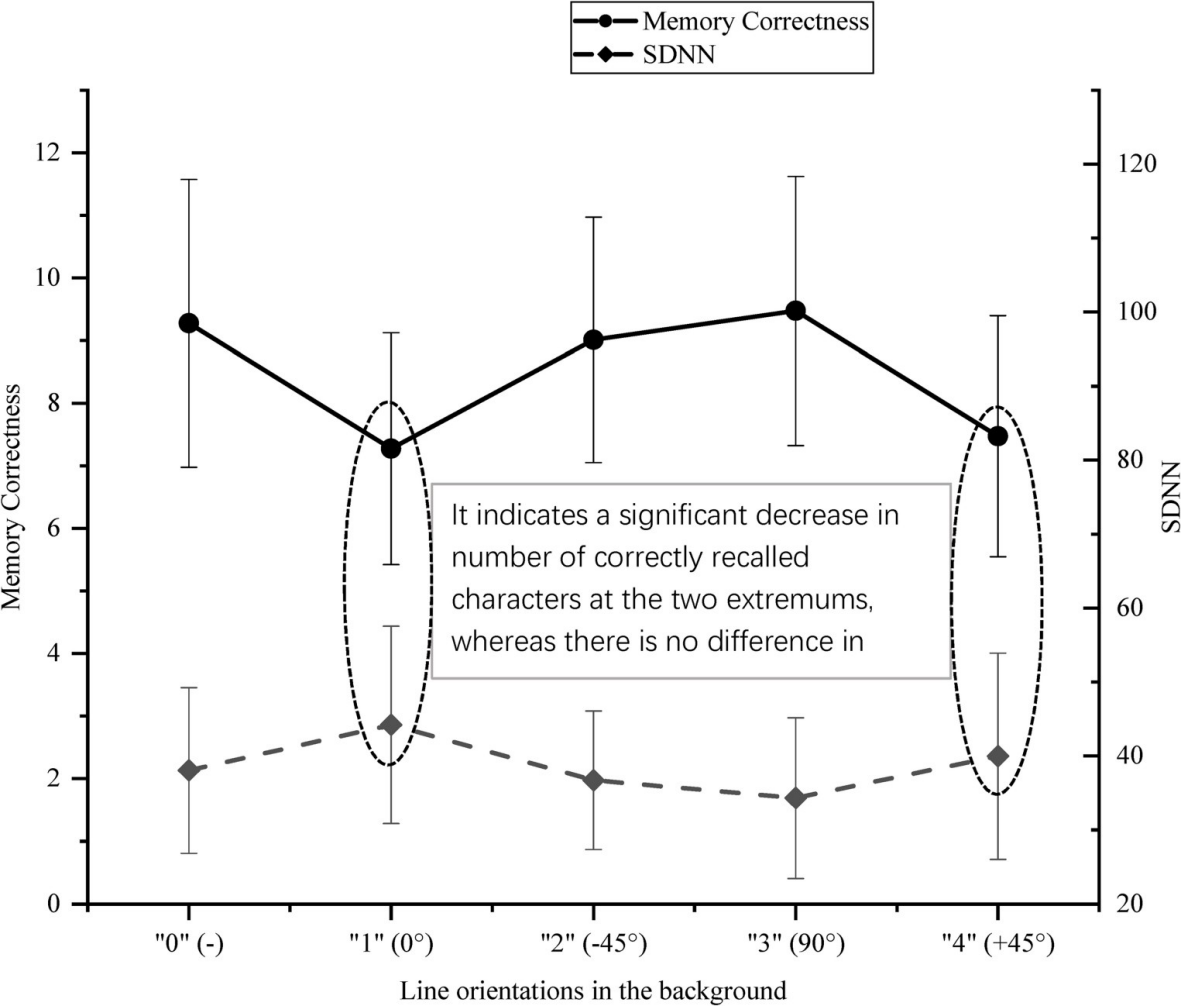
The mean values of correctly memorized foreground characters and users’ SDNN in different background line orientations.

Participants’ memory was evaluated by the number of correctly recalled characters that assistants received from each participant. The mean values of correctly recalled characters of the picture s’ foreground information are shown in Fig 3. It indicated that correctness was higher in conditions blank (-), -45°, and 90° than that in other two conditions. There were significant differences between the conditions blank (-) and 0°, blank (-) and +45°, 0° and -45°, 0° and 90°, -45° and +45°, and 90° and +45°, since the *p* values were all < 0.001 (*df* = 4, F = 23.532), and effect size was large (Partial *η^2^ =* 0.173).

### SDNN

Participants’ emotion was measured by SDNN. Fig 3 shows the means of participants’ SDNN. For the SDNN (*df* = 4, F = 2.864, *p* = 0.025, Partial *η^2^* = 0.073), mean values were lower in conditions blank (-), -45°, and 90° than that in the other two conditions. The differences between the conditions 0° and blank (-) (*p* = 0.049), 0° and -45° (*p* = 0.017), and 0° and 90° (*p* = 0.002) were significant, and the effect size was medium (Partial *η^2^* = 0.073); and there was no difference between other conditions.

Fig 3 also suggests the changing trends of memory and emotion: the mean values of correctly recalled characters arrived at a valley when the values of SDNN were at a peak.

## Discussion

We hypothesized that the orientations of lines in the background of an image impacted human’s emotion and memory subconsciously. By performing an experiment in a laboratory, where four pictures with distinct background line orientations and one with no background lines were presented to participants in varying sequences, with other control variables being constant (character difficulty, line width, background color, etc.), we measured users’ number of characters of the textual contents correctly recalled and their HRV values. During the experiment, only two out of 92 participants thought that the variations of the background line orientations affected their memory, which indicated that for most participants, memory performance was impacted subconsciously by environmental elements like background image information. Therefore, the results from the experiment are as follows.

Line orientations in the background of an image had a significant effect on users’ memory in a subconscious state: vertical and 45° leftward leaning lines did not decrease users’ memory, and no significant difference in memory was found between these two states and the blank background, while horizontal and 45° rightward leaning lines weakened the memory effect. The effect size (Partial η^2^ = 0.173 > 0.138) was large, which confirms the hypothesis that when a background features a pattern of equidistant lines, the line orientation has a certain subconscious influence on a user’s memory. Meantime, users’ HRV measurement showed negative similar trend as that of memory: the number of correctly recalled characters arrived at a valley when the SDNN was at a peak.

First, the results showed that memory decreased on horizontal condition while no influence on vertical condition comparing with blank background. It indicates that the memory differed in vertical from horizontal, which confirms previous research that the estimation of length differs significantly between vertical lines and horizontal lines [11], although people can perceive horizontals and verticals more easily than other orientations [22, 23].

Second, an asymmetric influence was found within the 45° leftward leaning lines (-45°) and 45° rightward leaning lines (+45°): memory decreased on rightward lined (+45°) background but was not affected by leftward lined (-45°) background comparing with blank condition. The asymmetry influence by leftward and rightward leaning lines could largely lie in the reading habit because all participants were left-to-right readers in this study. This is consistent with that reading habit has influence on users asymmetric perceptions and cognitions as attention [31], judgement [33, 34], and other behaviors [29, 35, 36]. However, it needs further research that whether an opposite result could be achieved from right-to-left readers.

In addition, the results showed that emotion and memory have negative similar trend, so that we speculate that line orientations resulted in different degrees of emotional arousal, since lines convey emotion [14], and horizontal/vertical lines and oblique lines indicate distinct affections respectively [15]. The emotion probably correlates with visuospatial bias [19].

## Conclusion

In conclusion, this study explored the impact of orientations of lines in the background of an image on human emotion and memory. The results showed that vertical (90°) and 45° leftward leaning lines (-45°) did not weaken users’ memory, and no significant difference in memory was found between these two states and the blank background, while horizontal (0°) and 45° rightward leaning lines (+45°) weakened the memory effect significantly. Meanwhile, the emotion and memory showed negative similar trend. These findings are meaningful for 2D graphic design, such as webpage and poster design, since it is crucial to impress users in a short period of time in this information society. Different line orientations can be used for different purposes, including to create appropriate memorable content.

However, there are still some problems that need further discussions. First, it is worth deeper exploration whether and how memory connects with emotion under the impact of line directions since the results of the two measurements showed negative similar trend. Prior researches showed inconsistent conclusions between emotion and memory [26–28], while in this study, the cause of emotion and its subsequent influence were unclear and need in-depth investigation. Second, what if the presented picture size was increased or decreased since size is a key issue in 2D visual design? It is therefore also worth considering whether the conclusions can be extended to three-dimensional designs, such as building orientation, furniture design, in-store goods arrangement, or exhibitions in future research. Moreover, it needs to include the impacts of line density, width, or shape etc. Third, how line orientations impact on cognitive performances of other languages or symbols, e.g., English words, icons, Latin letters, remains unknown and needs further explorations in next studies. Additionally, it is interesting to know what the emotion and memory effect would be if participants acknowledged the effect of background line orientations before the experiment.

Despite these limitations, this study concludes that designers should take the cognitive effects of line orientations, as well as aesthetic features, into account.

## Supporting information

S1 Table The English translation of Chinese stimuli characters. (PDF)

S1 Data The data from this study. (PDF)
