# Supplementary material for: The subconscious impact of line orientations in background images on memory of Chinese written characters
Source: PLoS One. 2022 May 31;17(5):e0269255. doi: 10.1371/journal.pone.0269255 (PMC9154177; doi:10.1371/journal.pone.0269255)
Supplement: S1 Table — (PDF) [file pone.0269255.s001.pdf]

S1 Table. The English translation of Chinese stimuli characters

**Picture 1**						
乘	同	荣	每	脸	英	简
Multiply	Same	Bloom	Each	Face	English	Simple
高	律	短	油	召	点	力
High	Law	Short	Oil	Call	Point	Power
养	时	升	踪	骨	北	盘
Adoption	Time	Increase	Trace	Bone	North	Plate
徐	清	美	通	怀	弟	画
Slow	Clear	Short for America	Through	Brace	Brother	Draw
**Picture 2**						
狠	面	启	巨	间	关	体
Ruthless	Face	Start	Very	Intra	Close	Body
易	临	攻	红	悄	近	云
Easy	Near	Tap	Red	Quietly	Close	Cloud
柴	赤	收	月	想	周	辽
Firewood	Red	Receive	Month	Think	Week	Far
旦	法	那	妥	烦	贡	舒
Day	Law	That	Good	Trouble	Contribute	Stretch
**Picture 3**						
节	辆	毛	听	训	及	杂
Node	Vehicle	Hair	Listen	Lessen	And	Mixed
手	见	才	甩	足	迭	贪
Hand	See	Just	Throw	Foot	Overlap	Greed
规	双	称	其	顿	另	肖
Ruler	Double	Weight	It	Pause	Other	Resemble
炸	预	寺	迫	会	昂	申
Explode	Predict	Temple	Forced	Will	Raise	Apply
**Picture 4**						
劣	位	底	思	招	弃	青
Bad	Position	Bottom	Think	Enroll	Throw	Green
尚	动	以	年	外	匠	语
Still	Move	With	Year	Outside	Craftsman	Speak
皮	各	如	余	控	名	闪
Skin	Each	For example	Remain	Control	Name	Flash
财	阻	分	须	从	服	休
Wealthy	Stop	Score	Must	From	Clothes	Rest
**Picture 5**						
勾	脏	者	式	咨	科	停
Hook	Dirty	Person	Pattern	Ask	Science	Stop
身	优	号	买	因	区	川
Body	Good	Number	Purchase	Reason	District	Mountain
饰	袖	尺	负	返	刃	吕
Decoration	Sleeve	Ruler	Negative	Back	Knife	A surname
宣	浓	独	搜	绿	醋	送
Broadcast	Thick	Alone	Search	Green	Vinegar	Give
