# Supplementary material for: The subconscious impact of line orientations in background images on memory of Chinese written characters
Source: PLoS One. 2022 May 31;17(5):e0269255. doi: 10.1371/journal.pone.0269255 (PMC9154177; doi:10.1371/journal.pone.0269255)
Supplement: S1 Data — (PDF) [file pone.0269255.s002.pdf]

No.		"0"(Blank)		"1"(0°)		"2"(-45°)		"3"(90°)		"4"(+45°)		Subjective responses		
Participar	Gender	memorized	SDNN	memorized	SDNN	memorized	SDNN	memorized	SDNN	memorized	SDNN	Noticed lines?	Noticed lines variation?	Line orientations influence memory?
1	0	11	33	7	36	14	39	9	45	9	47	1	1	
2	0	7	56	6	35	4	37	8	30	7	60	1	1	
3	0	11	19	6	32	10	20	10	19	10	26	1	1	
4	1	8	37	4	43	5	41	4	53	3	36			
5	1	9	17	7	28	11	24	8	14	6	24	1	1	
6	1	8	31	3	35	7	23	7	27	6	28	1	1	
7	1	8	41	7	39	7	36	6	37	6	68			
8	1	9	28	6	31	7	25	7	26	6	31	1	1	
9	0	15	55	10	82	10	55	11	54	9	78	1	1	
10	0	12	42	7	42	9	33	12	32	10	30	1	1	
11	0	11	25	7	40	12	23	11	22	7	27			
12	0	10	26	5	37	8	34	7	21	4	32	1	1	
13	0	9	37	9	37	7	31	7	26	7	25	1	1	
14	1	10	39	7	55	8	39	10	44	7	52			
15	1	13	48	7	53	10	55	11	40	5	47	1	1	
16	1	8	51	1	65	11	53	12	23	14	23			
17	1	11	53	4	72	7	55	8	45	6	45	1	1	
18	1	9	37	8	54	8	37	5	34	9	41			
19	1	10	38	9	33	9	32	11	30	11	39	1	1	
20	1	9	31	8	36	6	28	10	36	7	44	1	1	1
21	0	12	23	8	64	7	33	13	33	10	26	1	1	
22	0	14	47	11	60	12	41	13	64	9	37	1	1	
23	0	12	43	10	35	11	33	8	31	10	59	1	1	
24	0	10	30	9	28	8	40	9	27	4	30			
25	1	14	39	6	38	9	41	12	38	6	39	1	1	
26	1	9	60	5	45	8	42	7	39	7	52			
27	1	9	36	11	44	11	41	6	40	4	32			
28	0	9	52	9	53	8	43	10	40	7	57	1	1	
29	0	8	43	7	41	9	29	10	32	8	39	1	1	
30	0	9	24	6	33	7	38	10	27	7	25	1	1	
31	0	9		8		9		9		7				
32	0	6		11		7		8		7		1	1	
33	0	8		8		8		11		9				
34	1	6		9		8		10		7		1	1	
35	1	5		8		6		4		5		1	1	
36	1	6		7		8		12		7		1	1	
37	1	7		8		7		8		6				
38	1	13		9		9		7		7				
39	0	7		9		8		12		5		1	1	1
40	0	9		8		10		13		8				
41	1	9		7		10		8		6		1	1	
42	1	11		7		7		11		6				
43	0	13		8		10		13		9				
44	0	9		4		9		12		8		1	1	
45	0	11		12		12		12		11		1	1	
46	0	9		8		9		9		9		1	1	
47	0	5		6		9		9		7		1	1	
48	1	9		6		8		8		7		1	1	
49	1	7		5		7		6		9				

50	1	11	6	6	9	10		
51	1	9	6	9	7	9	1	1
52	1	8	4	8	9	6	1	1
53	0	7	7	9	9	10		
54	0	7	6	7	8	4	1	1
55	0	14	8	11	13	9		
56	0	10	7	9	12	7	1	1
57	0	9	5	9	10	5	1	1
58	0	4	5	6	8	6	1	1
59	0	9	8	13	11	6		
60	1	12	10	12	14	10	1	1
61	1	6	7	10	9	8		
62	1	4	5	8	6	7		
63	1	8	8	10	10	7		
64	1	5	8	14	9	11	1	1
65	0	9	10	13	11	9	1	1
66	1	8	8	7	7	8	1	1
67	0	8	6	8	9	5		
68	1	9	7	7	11	10		
69	1	10	7	10	11	6	1	1
Unqualifie	0	16	8	18	14	10	1	1
70	0	9	8	8	10	7	1	1
71	0	7	9	10	12	5		
72	0	14	5	7	10	6	1	1
73	1	8	6	9	8	8		
74	1	8	7	8	7	7	1	1
75	1	10	7	11	13	7		
76	1	9	8	11	11	7		
77	0	10	9	10	9	10	1	1
78	0	7	8	9	8	7		
79	0	12	5	12	11	9	1	1
80	1	7	8	10	8	8	1	1
81	1	10	7	10	10	8		
82	1	10	7	9	11	10	1	1
83	0	11	7	12	10	5	1	1
84	0	11	9	10	9	8		
85	1	8	6	8	9	7	1	1
86	0	7	8	10	8	6		
87	0	11	7	8	9	7	1	1
88	1	11	10	10	11	11	1	1
89	1	11	7	8	8	7	1	1
90	1	12	9	11	13	8		
91	0	11	9	12	10	8		
